# Sulphur-Bridged BAl_5_S_5_^+^ with 17 Counting Electrons: A Regular Planar Pentacoordinate Boron System

**DOI:** 10.3390/molecules26175205

**Published:** 2021-08-27

**Authors:** Yuhan Ye, Yiqiao Wang, Min Zhang, Yun Geng, Zhongmin Su

**Affiliations:** Institute of Functional Material Chemistry, Faculty of Chemistry & National & Local United Engineering Laboratory for Power Battery, Northeast Normal University, Changchun 130024, China; Yeyh442@nenu.edu.cn (Y.Y.); Wangyq538@nenu.edu.cn (Y.W.); gengy575@nenu.edu.cn (Y.G.); zmsu@nenu.edu.cn (Z.S.)

**Keywords:** planar pentacoordination, 17 counting electrons, boron multicoordination, sulfur bridging

## Abstract

At present, most of the reported planar pentacoordinate clusters are similar to the isoelectronic substitution of CAl_5_^+^, with 18 counting electrons. Meanwhile, the regular planar pentacoordinate boron systems are rarely reported. Hereby, a sulphur-bridged BAl_5_S_5_^+^ system with a five-pointed star configuration and 17 counting electrons is identified at the global energy minimum through the particle-swarm optimization method, based on the previous recognition on bridged sulphur as the peripheral tactics to the stable planar tetracoordinate carbon and boron. Its outstanding stability has been demonstrated by thermodynamic analysis at 900 K, electronic properties and chemical bonding analysis. This study provides adequately theoretical basis and referable data for its experimental capture and testing.

## 1. Introduction

Planar hypercoordinate motif is a significant field due to the structural novelty and the rule-breaking traditional recognition on covalent bonding among planar multiatoms. The atomic interaction in the plane configuration exists as a special category, which also possesses a profound impact on the understanding of 2D material structure and the corresponding functional properties. Hence, the plane pentacoordinate carbon (PPC) continues its development and research on the foremost basis of the plane tetracoordination [1]. In addition, understanding the stability exhibited by planar tetracoordinate structures also elucidates further approach to design plane multicoordinate systems [2,3]. Hoffmann et al. [4] firstly theoretically proposed to stabilize plane tetracoordinate carbon (PTC) structure [5,6,7] with electronic strategy [8] in 1970, and designed planar PTC structure with the lower energy, which attracted extensive attention. With the development of PTC in clusters, planar penta-, hexa- and hepta-coordinate carbon systems as well as other planar multicoordinate carbon (PMC) structures have also been proposed and reported. For example, CB_6_^2−^, CB_7_^−^, CB_8_^−^, etc., have been designed or synthesized theoretically or experimentally [9,10,11,12,13,14].

Due to the unique electronic deficient nature of boron element, planar multicoordinate boron (PMB) structures widely exist in some ionic states of boron clusters and boron-rich systems, which tend to form multicentric bonds between boron atom and other adjacent atoms in nature [15,16,17,18,19,20,21,22,23,24,25,26]. In 2004, the first global energy minimum (GEM) BCu_5_H_5_^−^ with *D_5h_* symmetry and bridged hydrogen was first proposed [27]. In 2005, some neutral all-B aromatic clusters with PMB were established according to topological resonance energy. Ref. [28] In 2008, Pei et al. theoretically proposed a series of planar tetra-, penta- and hexa-coordinated carbon-boron mixed clusters [29]. In 2009, Yu et al. proposed the borane cation B_6_H_5_^+^ with *D_5h_* symmetry and bridged hydrogen, which is another instance of PPB at the GEM and exhibits good aromaticity [30]. Meanwhile, many instances containing PPC are also reported. In 2010, Jimenez-Halla et al. reported the formation of isoelectronic CAl_4_Be and CAl_3_Be_2_^−^ clusters by replacing Al atoms in CAl_5_^+^ with Be and regulating the related charges, where the central carbon acts as the σ receptor and the σ bond possesses a feedback synergistic effect from the C 2p_z_ orbital to the conjugated π bond [31]. In 2015, Guo et al. proposed that CAl_2_Be_3_^2−^ could realize VDE stabilization through Li^+^ and divalent anion complexation [32]. In 2015, Grande-Aztatzi et al. proposed that CBe_5_Li*_n_^n^*^−4^ (*n* = 2–5) could reduce the electrostatic effect by combining with Li^+^ ions [33]. Ding et al. proposed the CAl_4_X^+^ cluster, CAl_4_MX_2_ cluster [34] and other transition metal bond cooperation [35]. Until now, most of the planar pentacoordinate clusters fit the isoelectronic substitution of CAl_5_^+^, namely, the 18 counting electron rule (the total valence electron including the central atom and its adjacent coordinate atoms), which plays a decisive role in providing the corresponding stability [36]. In the latter, researchers found that generally the thermodynamic preference of the 17e/18e counting rule is over the 15e/16e rule.

The GEM structure of B_6_S_5_ with a plane pentagonal configuration and 18 counting electrons has been identified by our group [37]. Due to the co-existence of π bridge bond and σ bond, the B-S-B-bridged connection effectively realized the combination of electronic strategy and mechanical strategy [37]. Before that, the sulphur-bridged PTC and tetracoordinate boron (PTB) structures were also studied by our group [38,39]. All these studies demonstrate that S atom, as a special bridging element, could help to obtain stabilized planar multicoordinate systems. A five-pointed star configuration of XAl_5_S_5_*^n^* (X = C, B; *n* = 0, +1, +2) was considered in this study, with B and C as the central atom and S as the peripheral bridged atoms. Through the structure search and the subsequent reconfirmation, the BAl_5_S_5_^+^ structure was finally identified as an excellent system with a five-pointed star configuration at its GEM and 17 counting electrons. Its outstanding stability has been further verified by the corresponding orbital analysis, thermodynamic analysis, and so on. The details are provided in the following parts.

## 2. Results and Discussion

### 2.1. Structure and Stability of XAl_5_S_5_^n^ (X = C, B; n = 0, +1, +2) Clusters

The initially generated structures were all identified at B3LYP/6-31G for XAl_5_S_5_*^n^* (X = C, B; *n* = 0, +1, +2) clusters through the particle-swarm optimization method. The obtained structures with no imaginary frequency were further optimized at B3LYP/def2-TZVP level. Finally, single-point calculations were carried out for the four isomers with the lowest energies at the CCSD/6-31G(d) level and with ZPE corrections at the same level.

**Figure 1:**
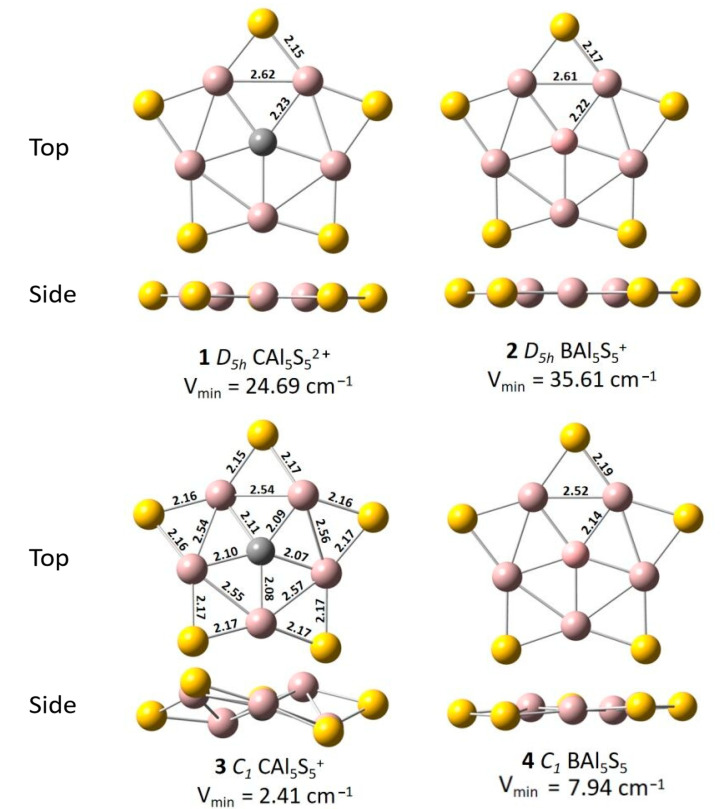
The Optimized Structures and Bond Lengths for XAl_5_S_5_ (X = C, B; *n* = 0, +1, +2) clusters and their lowest frequencies at CCSD/6-31G(d) level.

The GEM structures of four systems with PPC or PPB are all shown in Figure 1, and their other low-lying structures are shown in Figure S1 in the Supplementary Materials. The energy of PPC *D_5h_* CAl_5_S_5_^2+^ with 17 counting electrons is 14.68 kJ/mol lower than that of the second-lowest energy structure. Meanwhile, the energy of PPB *D_5h_* BAl_5_S_5_^+^ with 17 counting electrons is 28.90 kJ/mol lower than that of the second-lowest energy structure. These two systems at their GEM structures with 18 counting electrons are also with quasi-planar pentacoordinate central atom. However, their symmetries both drop to *C_1_*. Other information about the low-lying structures of these systems is all given in Figure S1 in the Supplementary Materials.

**Figure 2:**
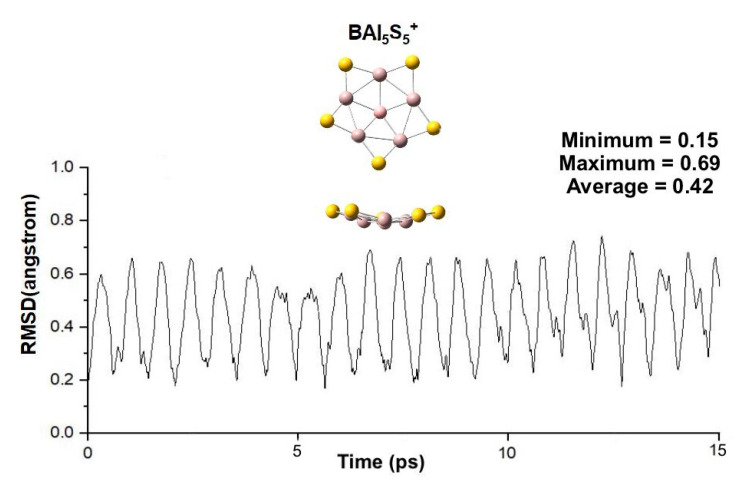
Root-mean-square deviations (RMSD) of BAl_5_S_5_^+^ during Born–Oppenheimer molecular dynamics (BOMD) simulations at 900 K. The final snapshot of the structure is also shown here.

Then, Born–Oppenheimer molecular dynamics (BOMD) simulations [40] of four GEM structures were performed over 15 ps at ambient temperature, 600 K and 900 K for the evaluation on their dynamic stability. The PPB *D_5h_* BAl_5_S_5_^+^ with 17 counting electrons can keep its structural integrity even at 900 K (Figure 2), which is extremely robust against isomerization and decomposition. Its RMSDs fluctuate relatively small, suggesting its good kinetic stability. In contrast, The PPC *D_5h_* CAl_5_S_5_^2+^ with 17 counting electrons can keep its structural integrity at 600 K (Figure S2 in the Supplementary Materials). The other two systems with 18 counting electrons also can keep their structural integrity at 298 K (Figure S2 in the Supplementary Materials). These data demonstrate: firstly, *D_5h_* BAl_5_S_5_^+^ with the highest stability; secondly, the systems with 17 counting electrons are more stable than those with 18 counting electrons in our systems.

### 2.2. Electronic Properties and NBO Analysis on CAl_5_S_5_^+^, CAl_5_S_5_^2+^, BAl_5_S_5_, BAl_5_S_5_^+^

To investigate and understand the reason why BAl_5_S_5_^+^ possesses the best stability among these systems, the electronic properties and bonding analysis on CAl_5_S_5_ and BAl_5_S_5_ with 17 and 18 counting electrons are discussed here. Table 1 lists their NPA and WBI bond orders obtained from NBO analysis. Through further structural analysis, it can be found that the central C and B are negatively charged, the aluminum is positively charged, and the peripheral sulfur is negatively charged. The negative–positive–negative distribution of electrons is beneficial to their stability. According to bond level analysis, the Al-Al Wiberg bond level is very weak (the WBI_Al-Al_ values are all below 0.14), indicating that their interatomic bonding is very weak. The key factor to stabilize the PPC and PPB atoms is the C-Al and B-Al bonding, as well as the stability of the Al-S-Al bond in the periphery. This also indicates that S, as a bridged atom, plays an important role in maintaining the stability of the plane structure.

The total WBI bond levels of C atoms in electron CAl_5_S_5_^2+^ (17e) and 18 electron CAl_5_S_5_^+^ (17e) are 2.06 and 2.43, respectively. Similarly, the total WBI bond levels of B atoms in electron BAl_5_S_5_^+^ (17e) and 18 electron BAl_5_S_5_ (18e) are 2.80 and 3.36, respectively. This indicates that the interaction between central B atom and adjacent Al atoms is much stronger than that between central C atom and adjacent Al atoms. Meanwhile, the dynamic stability of BAl_5_S_5_^+^ is best, although its WBI total value is not the highest one.

**Figure 3:**
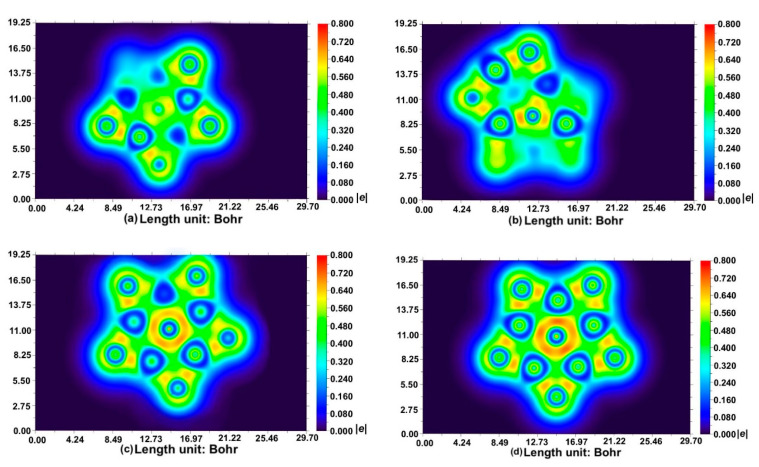
Localized orbital locator (LOL) analysis of (**a**) CAl_5_S_5_^+^, (**b**) CAl_5_S_5_^2+^, (**c**) BAl_5_S_5_, (**d**) BAl_5_S_5_^+^.

Localized orbital locator (LOL) [41] analysis can well reflect the electron distribution in the whole system (Figure 3). It is clearly easy to see that the electron distribution is symmetrically distributed in BAl_5_S_5_^+^. No region with electron density is over 0.6e there, indicating that electron repulsion is relatively small. To clarify, the difference of electron repulsion caused the last counting electron. The uneven bonding and unevenly distributed electron in other systems must result in the reduction in the stability. Furthermore, the existence of the higher electron density in the small area also produces stronger electron repulsion. In addition, the electron distribution and the corresponding energy of the six highest-occupied π MOs in BAl_5_S_5_^+^ and BAl_5_S_5_ are shown in Figures S3 and S4 in the Supplementary Materials, which is a very important factor to maintain the planar structure. Clearly, the similar degenerate π MOs exists, but the energies of the orbital energies of BAl_5_S_5_ are very high compared to those α energies f BAl_5_S_5_^+^. These also can elucidate why BAl_5_S_5_^+^ is the best one among all the investigated systems. That is consistent with the previous BOMD analysis (Figure 2).

### 2.3. Chemical Bonding on the PPB BAl_5_S_5_^+^ Cluster

**Figure 4:**
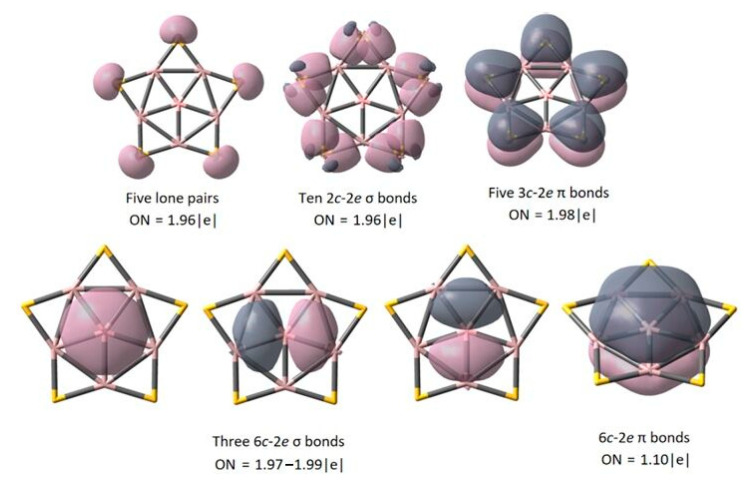
AdNDP analysis of *D_5h_* BAl_5_S_5_^+^.

The internal bonding mode and electron distribution characteristics can be intuitively understood by semi-localized AdNDP analysis. After the previous stability exploration, the AdNDP analysis on BAl_5_S_5_^+^ is executed (Figure 4). The analysis clearly shows the lone pair of five S atoms, the two-center–two-electron (2c–2e) σ bond between Al-S atoms and the three-center–two-electron (3c–2e) π bond between Al-S-Al atoms. The 5π bonds fit the rule of Hückel aromatics (2 × 2 + 1), which is one of the factors that stabilize the whole structure. In addition, the central atom B of the planar pentacoordination is stabilized by five delocalized bonds: three delocalized 6c–2e σ bonds and one delocalized π single electron in the 6c–2e bond (electron-occupied number is about 1.1e). The 3σ bonds indicate the σ contribution to the central atom B, while the inner single-electron π bond overlaps weak with the outer 5π bonds. The semi-localized AdNDP analysis showed that the electron repulsion in the system was very weak, which made BAl_5_S_5_^+^ exhibit good stability at 17 electrons.

**Figure 5:**
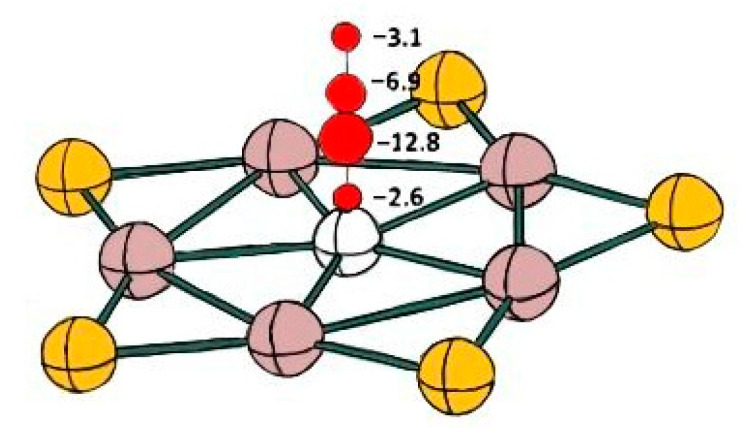
NICS value analysis of *D_5h_* BAl_5_S_5_^+^.

In order to confirm the conclusion of aromaticity obtained by AdNDP analysis, we calculated the NICs values for the structure of BAl_5_S_5_^+^. The NICS value over central B atom at 1 Å and 2 Å are both negative (−12.80 ppm and −3.13 ppm), indicating its excellent aromaticity. The NICS values at other positions are also shown in Figure 5. The aromatic existence in BAl_5_S_5_^+^ makes its structure stable, which provides a further explanation for the previous kinetic and thermodynamic stability.

## 3. Computational Methodology

The global minimum structural search of all the XAl_5_S_5_*^n^* (X = C, B; *n* = 0, +1, +2) clusters were conducted with the particle-swarm optimization (PSO) method [42], as implemented in the CALYPSO [39] code. More than 1000 stationary points were generated and searched for each of the species. All the calculations, including optimization and the corresponding analysis, were executed using the Gaussian 09 package [43]. Restricted calculation is used for closed-shell systems, and unrestricted calculation is used for open-shell systems and odd-electron systems, and the energy differences caused by spin multiplicity are also considered. These PPB species, together with other low-lying states, were re-optimized and the harmonic vibrational frequencies analysis at the hybrid B3LYP functional in connection with def2-TZVP basis set to further ensure they are the ground states [44]. Finally, these GEM clusters of XAl_5_S_5_*^n^* were analyzed using Natural Bond Orbital (NBO) 3.0 in Gaussian 09 for obtaining natural population analysis (NPA), Wiberg bond indices (WBIs). Adaptive natural density partitioning (AdNDP) [44] analyses were operated at the same level using the Multifwn3.3.8 program [41].

## 4. Conclusions

A BAl_5_S_5_^+^ system with a five-pointed star configuration and 17 counting electrons is identified at the global energy minimum.

The sulphur-bridged planar structure of BAl_5_S_5_^+^ with a five-pointed star configuration at the global energy minimum is identified through the particle-swarm optimization method and possesses the best stability among the investigated systems of XAl_5_S_5_*^n^* (X = C, B; *n* = 0, +1, +2). By calculating BOMD, BAl_5_S_5_^+^ endures the best stability at 900 K. The following analysis on the electronic property and chemical bonding clearly interprets the contribution from peripheral S-bridged connection and the 17 counting electrons. This study provides adequately theoretical basis and referable data for its experimental capture and testing.

## Figures and Tables

**Table 1:** Important NPA charges and Wiberg Bond Indices (WBIs) of CAl_5_S_5_^+^, CAl_5_S_5_^2+^, BAl_5_S_5_, BAl_5_S_5_^+^.

	CAl_5_S_5_^+^ (18e)	CAl_5_S_5_^2+^ (17e)		BAl_5_S_5_ (18e)	BAl_5_S_5_^+^ (17e)
Q_C_	−2.50	−1.87	Q_B_	−2.56	−0.63
Q_Al_	1.42/1.43/1.44	1.40	Q_Al_	1.31	0.66
Q_S_	−0.72/−0.73/−0.74	−0.63	Q_S_	−0.80	−0.34
WBI_C_	2.43	2.06	WBI_B_	3.36	2.80
WBI_Al_	2.47–2.51	2.51	WBI_Al_	2.66	2.61
WBI_S_	1.99–2.02	2.10	WBI_S_	1.92	1.99
WBI_C-Al_	0.40–0.45	0.36	WBI_B-Al_	0.60	0.50
WBI_Al-Al_	0.10	0.10	WBI_Al-Al_	0.14	0.12
WBI_Al-S_	0.89–0.93	0.96	WBI_Al-S_	0.85	0.91

## Data Availability

Data are contained within this article.

## Supplementary Materials

The following are available online, Figure S1: The optimized structures of the four lowest isomers of PPC/B and quasi-PPC systems at B3LYP/def2-TZVP and the corresponding single-point energies at CCSD/6-31g(d) and the lowest frequency, Figure S2: Born–Oppenheimer molecular dynamics (BOMD) simulations at different temperatures and their root-mean-square deviations (RMSD) for four systems. Figure S3: The α electron profile and the corresponding energy of the 6 highest-occupied π MOs of BAl_5_S_5_^+^. Figure S4: The electron profile and the corresponding energy of the 6 highest-occupied π MOs of BAl_5_S_5_. Table S1: The important information on the GEM structures of PPC/B and quasi-PPC systems at B3LYP/def2-TZVP level.
