# Supplementary material for: Sulphur-Bridged BAl5S5+ with 17 Counting Electrons: A Regular Planar Pentacoordinate Boron System
Source: Molecules. 2021 Aug 27;26(17):5205. doi: 10.3390/molecules26175205 (PMC8433653; doi:10.3390/molecules26175205)
Supplement: Supplementary file 1 [file molecules-26-05205-s001.zip › molecules-1337588-supplementary.pdf]

SUPPLEMENTARY MATERIALS

Sulphur-bridged $BAl_5S_5^+$ with 17 counting electrons: a regular planar pentacoordinate boron system

Yuhan Ye, Yiqiao Wang, Min Zhang*, Yun Geng and Zhongmin Su

Institute of Functional Material Chemistry, Faculty of Chemistry & National & Local United Engineering Laboratory for Power Battery, Northeast Normal University, Changchun 130024, P.R. China.

E-mail: mzhang@nenu.edu.cn

Figure S1. The optimized structures of four lowest isomers of PPC/B and quasi-PPC systems at B3LYP/def2-TZVP and the corresponding single point energies at CCSD/6-31g(d) and the lowest frequency. (Relative energies are listed in kcal mol^{-1} and the minimum frequencies are listed in cm^{-1})

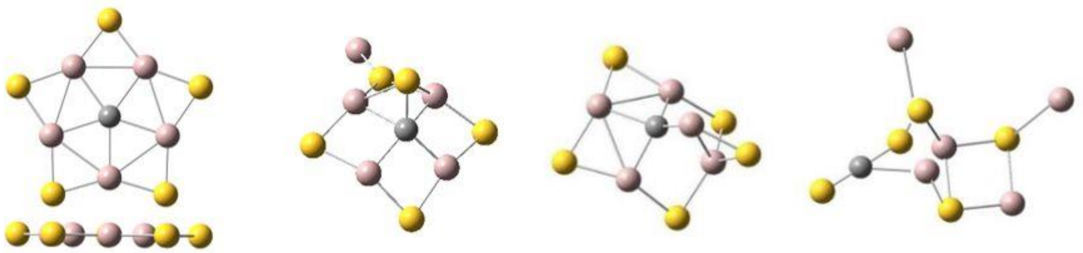

**1** $CAl_5S_5^{2+}$ D_{5h}	**1B** C_S	**1C** C_S	**1D** C_1
ΔE = 0.00	ΔE = +14.68	ΔE = +30.23	ΔE = +51.09
V_{min} = 24.69	V_{min} = 20.41	V_{min} = 54.49	V_{min} = 8.99

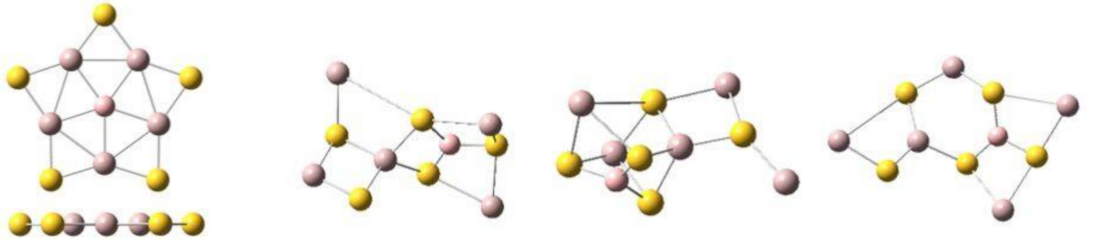

**2** $BAl_5S_5^{+}$ D_{5h}	**2B** C_1	**2C** C_1	**2D** C_1
ΔE = 0.00	ΔE = +28.90	ΔE = +29.80	ΔE = +45.00
V_{min} = 35.61	V_{min} = 19.70	V_{min} = 17.39	V_{min} = 8.90

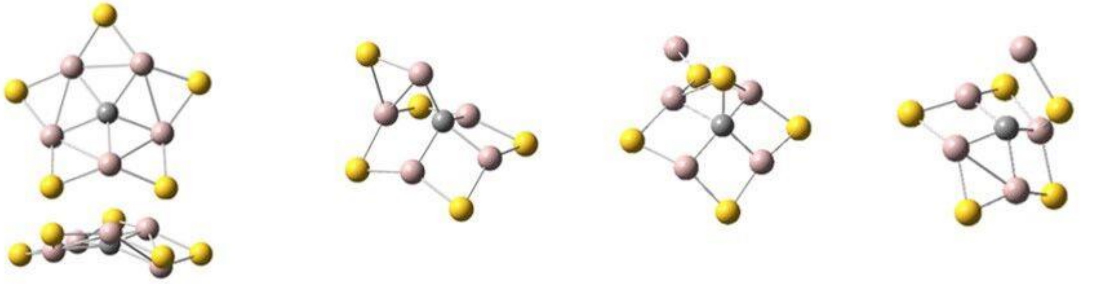

**3** $CAl_5S_5^{+}$ C_1	**3B** C_S	**3C** C_S	**3D** C_1
ΔE = 0.00	ΔE = +16.10	ΔE = +39.60	ΔE = +41.45
V_{min} = 2.41	V_{min} = 60.02	V_{min} = 27.06	V_{min} = 46.72

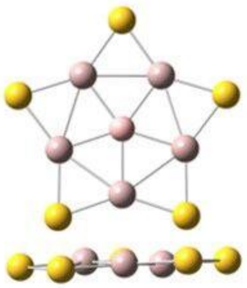

4 BAl_5S_5 *C_1*
ΔE = 0.00
V_{min} = 7.94

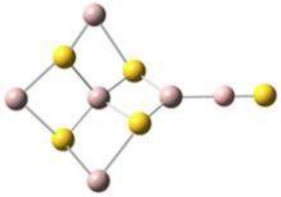

4B *C_1*
ΔE = +37.59
V_{min} = 22.63

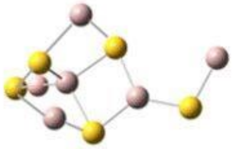

4C *C_1*
ΔE = +40.89
V_{min} = 18.85

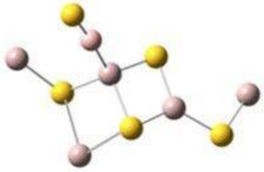

4D *C_1*
ΔE = +41.99
V_{min} = 13.26

Figure S2. Born-Oppenheimer molecular dynamics (BOMD) simulations at different temperatures and their Root-mean-square deviations (RMSD) for four systems.

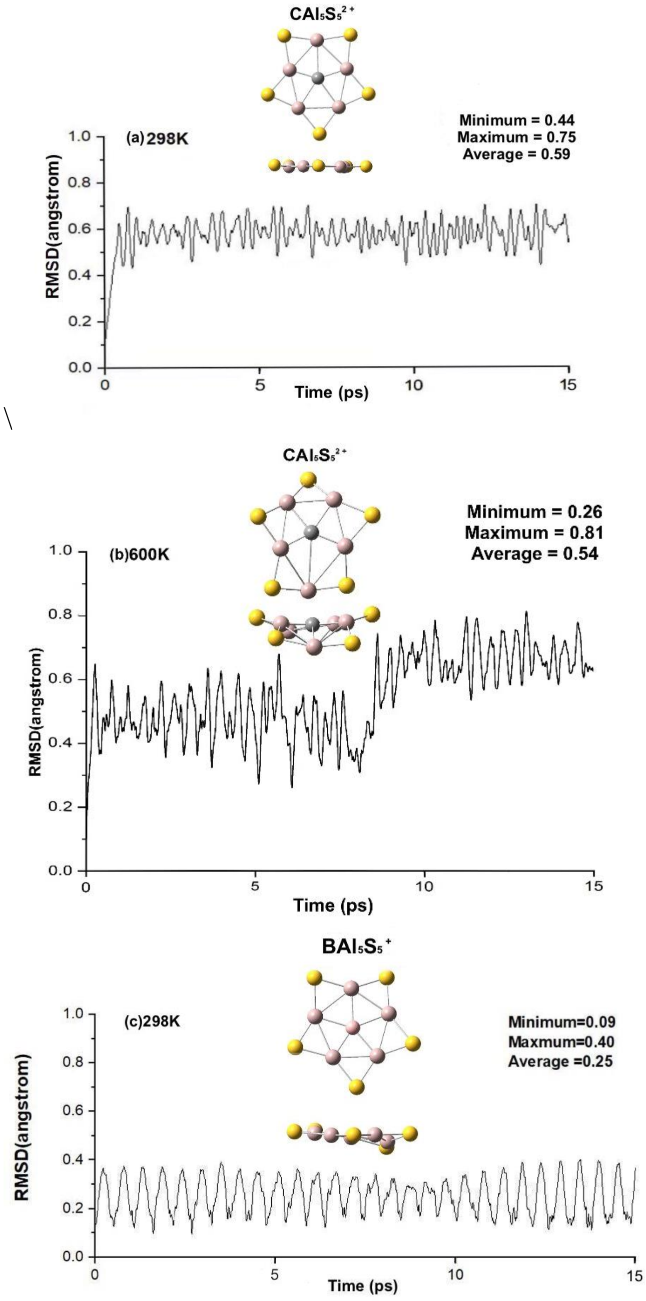

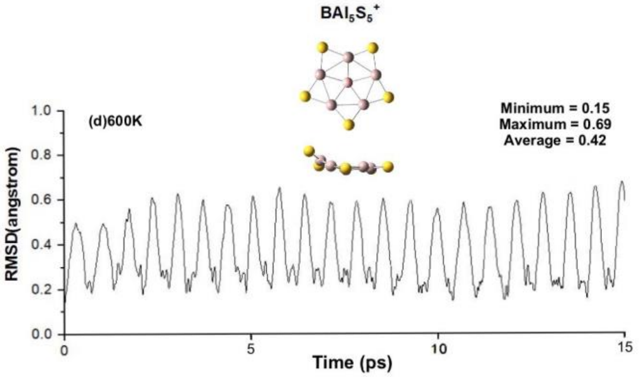

$BAl_5S_5^+$
(d)600K
Minimum = 0.15
Maximum = 0.69
Average = 0.42
RMSD(angstrom)
Time (ps)

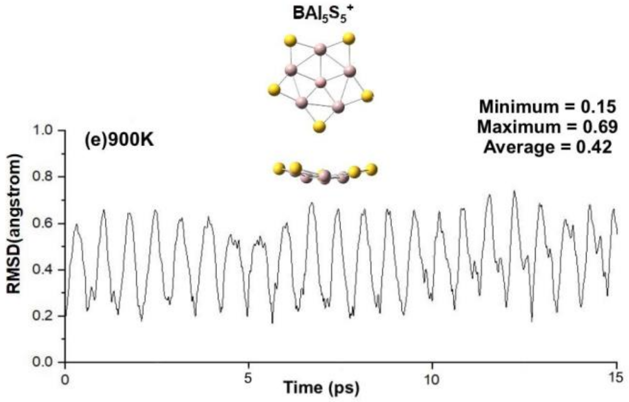

$BAl_5S_5^+$
(e)900K
Minimum = 0.15
Maximum = 0.69
Average = 0.42
RMSD(angstrom)
Time (ps)

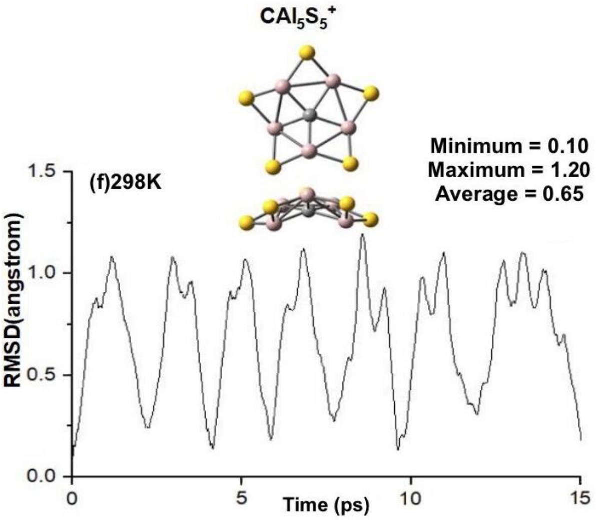

$CAl_5S_5^+$
(f)298K
Minimum = 0.10
Maximum = 1.20
Average = 0.65
RMSD(angstrom)
Time (ps)

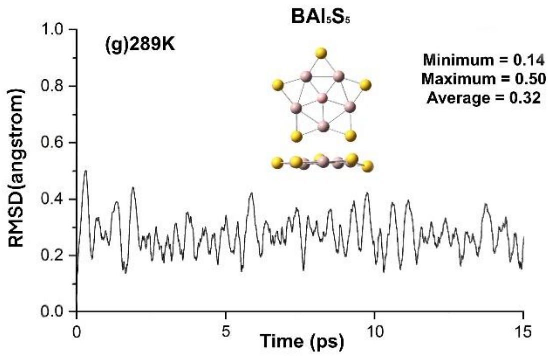
BAl5S5
(g)289K
Minimum = 0.14
Maximum = 0.50
Average = 0.32
RMSD(angstrom)
Time (ps)
1.0
0.8
0.6
0.4
0.2
0.0
0
5
10
15

Figure S3. The α electron profile and the corresponding energy of 6 highest occupied π MOs of $BAl_5S_5^+$.

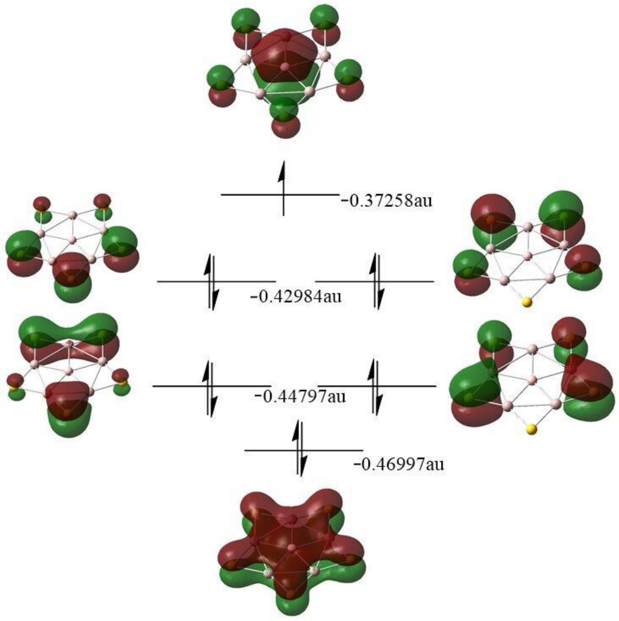

FigureS4. The electron profile and the corresponding energy of 6 highest occupied π MOs of BAl_5S_5.

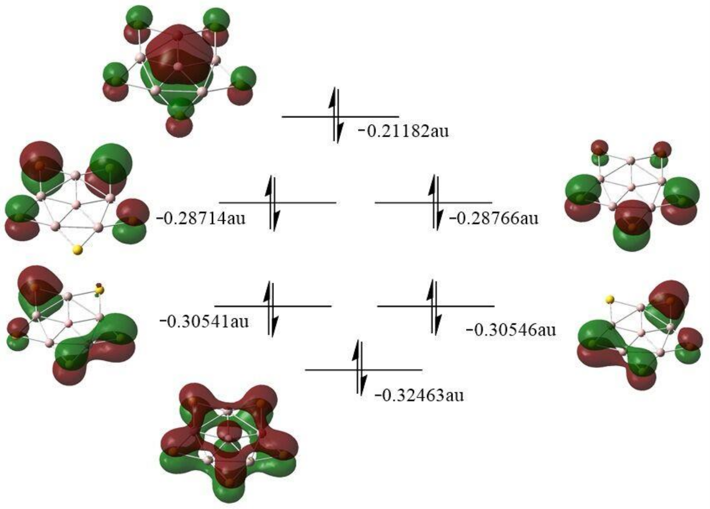

Table S1. The important information on the GEM structures of PPC/B and quasi-PPC systems at B3LYP/def2-TZVP level.

1. $CAl_5S_5^{2+}$

(D_{5h}, $^2A_2''$)

$E_{total\ with\ ZPE}$ = -3241.261471 Hartree Freq$_{min}$ = 24.6879 cm^{-1}

C	0.00000000	0.00000000	0.00000000
Al	0.00000000	2.23120000	0.00000000
Al	2.121997300	0.68947871	0.00000000
Al	-2.121997300	0.68947871	0.00000000
Al	-1.311466455	-1.80507872	0.00000000
Al	1.311466456	-1.80507871	0.00000000
S	2.058719183	2.83358386	0.00000000
S	-2.058719185	2.83358386	0.00000000
S	-3.331077613	-1.08233272	0.00000000
S	0.00000000	-3.50250227	0.00000000
S	3.331077613	-1.08233272	0.00000000

2. $BAl_5S_5^{+}$

(D_{5h}, $^2A_2''$)

$E_{total\ with\ ZPE}$ = -3228.429483 Hartree Freq$_{min}$ = 47.6385 cm^{-1}

B	0.00000000	0.00000019	0.00000000
Al	-0.00000004	2.21284859	0.00000000
Al	2.10454387	0.68380800	0.00000000
Al	-2.10454390	0.68380790	0.00000000
Al	-1.30067961	-1.79023179	0.00000000
Al	1.30067969	-1.79023173	0.00000000
S	2.07676105	2.85841668	0.00000000
S	-2.07676117	2.85841659	0.00000000
S	-3.36027004	-1.09181781	0.00000000
S	0.00000007	-3.53319684	0.00000000
S	3.36027009	-1.09181766	0.00000000

3. $CAl_5S_5^{+}$

(C_1, ^1A)

$E_{total\ with\ ZPE}$ = -3241.699475 Hartree Freq$_{min}$ = 8.2409 cm^{-1}

C	0.00183052	0.00607964	-0.00022898
Al	0.62539807	2.01974485	-0.00745236
Al	-1.72684059	1.14419377	-0.33295140
Al	2.06717006	-0.02933438	0.34989116
Al	0.61258798	-1.90045662	-0.56768049
Al	-1.57701658	-1.23129851	0.55742595

S	-1.09320251	3.20141134	-0.56184501
S	2.70658908	2.02847855	0.56153558
S	2.77231980	-1.94851426	-0.34555980
S	-0.99914694	-3.24135668	-0.01350604
S	-3.38770491	-0.04919371	0.35950640

4. BAl_5S_5

(C_1, ^1A)

$E_{total\ with\ ZPE}$ = -3228.685859 Hartree Freq$_{min}$ = 12.3348 cm^{-1}

B	0.00033891	-0.00026289	-0.00021126
Al	1.21666051	1.75701594	0.10837474
Al	-1.29226048	1.70066384	-0.13412062
Al	2.05382821	-0.61724278	-0.04214532
Al	0.04414126	-2.13846365	-0.04128160
Al	-2.02197418	-0.70228322	0.10781162
S	-0.07588540	3.52494250	-0.02281492
S	3.33015928	1.16351663	0.06089086
S	2.13304280	-2.80975896	-0.07471912
S	-2.01144390	-2.89706526	0.06119449
S	-3.37629990	1.01869901	-0.02337932
